# Effect of Cluster Nursing Based on Risk Management Strategy on Urinary Tract Infection in Patients With Severe Craniocerebral Injury

**DOI:** 10.3389/fsurg.2021.826835

**Published:** 2022-02-03

**Authors:** Hongbin Qiao, Jing Yang, Cui Wang

**Affiliations:** ^1^The Hospital Infection Management Department, Chongqing Southeast Hospital, Chongqing, China; ^2^The Department of Respiratory and Critical Care Medicine, Chongqing People's Hospital, Chongqing, China

**Keywords:** craniocerebral injury, catheter-related urinary tract infection, risk management, cluster nursing, effect

## Abstract

**Objective:**

To observe the effect of cluster nursing based on risk management strategy in the management of urinary tract infection in patients with severe craniocerebral injury.

**Methods:**

A total of 116 patients with severe craniocerebral injury who were admitted to our hospital from March 2019 to March 2021 were included. They were divided into the control group (58 patients) and the observation group (58 patients). The control group received routine nursing care and the observation group received cluster nursing based on risk management strategy. The incidence of catheter-associated urinary tract infection (CAUTI), the results of bacterial culture on the surface of the urinary catheter, the incidence of nursing risk events, the duration of placing the urinary catheter, the length of hospital stay, and hospital costs as well as the patient satisfaction score were compared between the two groups. The knowledge, attitude, and practice scale for prevention of catheter infection and the competence evaluation scale of nurses were used to evaluate the sense-control ability and core competence of the interveners.

**Results:**

The total incidence of CAUTI in the observation group was (6.90%) lower than that in the control group (20.69%) (*p* < 0.05). The bacterial culture results on the catheter surface of patients in the observation group before and after 6 and 12 h of catheter cleaning were better than those of patients in the control group (*p* < 0.05). The duration of indwelling urinary catheter, hospitalization time, and hospitalization expenses of patients in the observation group were lower than those of patients in the control group (*p* < 0.05). The incidence rate of nursing risk events in the observation group was (1.72%) lower than that in the control group (11.86%) (*p* < 0.05). The overall satisfaction score of patients and the control and core ability scores of nursing staff in the observation group were higher than those in the control group (*p* < 0.05).

**Conclusion:**

Cluster nursing based on risk management strategy can effectively reduce the incidence of nursing risk events and the probability of UTI in patients with severe craniocerebral injury, shorten the duration of indwelling urinary catheter and hospitalization.

## Introduction

Severe craniocerebral injury is a common disease in brain surgery, often caused by traffic accidents, falling from high altitude, and other accidents (1). In the recent years, with the increase of space distance and frequent traffic accidents, the incidence of severe craniocerebral injury is increasing year by year. After injury, patients usually have symptoms of different degrees such as dilated pupils, vomiting, nausea, pain, disorder of vital signs, and disturbance of consciousness. In severe cases, serious sequelae such as hemiplegia or even death may even occur, which threaten health and should be paid attention to (2, 3). Patients with severe craniocerebral injury are in critical condition and progressing rapidly. They usually need to have an operation. After the operation, patients need to stay in bed for a long time during hospitalization and leave a catheter to urinate. Indwelling catheter is a commonly used invasive operation method in clinic, especially for some patients with urinary incontinence and coma. It cannot only accurately observe and record the urine volume and the urine proportion of patients with severe craniocerebral injury, but also prevent surgical complications. At the same time, it can also treat dysuria and train the bladder function of patients (4, 5). However, some studies have pointed out that the longer the urinary catheter is used, the higher the probability of catheter-associated urinary tract infection (CAUTI) in patients. In the United States, about 70% of complicated UTIs can be attributed to the use of catheters. CAUTI is associated with an increase in the morbidity and mortality of hospital infection and it is the most common cause of secondary blood-borne infection (6). Improper catheterization will damage the urethra and bladder mucosa and long-term indwelling will weaken the function of detrusor. Urine calcium salt deposition will block the urinary catheter, resulting in urine leakage and damage to the natural defense barrier of the urinary tract, causing bacterial colonization or infection (7). At present, care of clinical nurses for indwelling urinary catheter is still based on Basic Nursing Science, but there are still differences in nursing measures such as selection of urinary catheter of different departments and materials, frequency of urine bag replacement, timing of catheter removal, and evaluation and rehabilitation of bladder function after catheter removal, which have resulted in long time of indwelling urinary catheter in some patients, increased probability of urethral injury and infection, and deepened doctor–patient contradiction (8, 9).

Risk management is a management science that refers to finding, evaluating, and seeking countermeasures for economic loss risks and its purpose is to reduce losses and legal proceedings (10). Medical risks are ubiquitous. Patients with severe craniocerebral diseases are in critical condition and accept various diagnosis and treatment operations including indwelling catheter. The risk of nosocomial infection is several times higher than that of other patients in general wards and most infections are catheter-related infections. Therefore, implementing risk management and controlling CAUTI have become the research focus of medical staff. Cluster nursing was first proposed by the American Health Research Institute and is a set of nursing interventions formulated to address nursing problems that have many clinical influencing factors and are difficult to solve. Since the concept of cluster nursing was put forward, because it is novel, systematic, and more effective, the international community has carried out applied research on clinical nursing of many diseases (11–13). This study has shown that the implementation of cluster nursing strategy can reduce the incidence of CAUTI in patients (14). Cluster nursing based on risk management strategy is not a simple bundle of some clinical nursing measures, but its formulation needs the support of evidence-based theory and clinical evidence. Through formulating an early warning system for the risk of a certain disease or adverse event, we can formulate personalized nursing measures and strengthen early warning and risk management for complications of patients, so as to improve nursing efficiency and reduce nursing risk. In this study, during the management of UTI in patients with severe craniocerebral injury, cluster nursing based on risk management strategy was implemented, which was compared with conventional nursing methods, in order to reduce the incidence of CAUTI in patients and provide reference for clinical indwelling catheter and follow-up nursing.

## Data and Methods

### General Information

A total of 116 patients with severe craniocerebral injury who were admitted to the hospital from March 2019 to March 2021 were included. Inclusion criteria were as follows: (1) patients who meet the criteria for severe craniocerebral injury and age ≥18 years; (2) the catheter needs to be indwelling; (3) the estimated duration of indwelling urinary catheter ≥5 days; and (4) families of patients could cooperate with relevant nursing work, research progress, and sign informed consent. Exclusion criteria were as follows: (1) infection occurred within 48 h after admission; (2) there was a history of anti-infection treatment in the last month; (3) the urinary catheter was indwelling before entering the group; (4) complicated with severe organ diseases such as heart, liver, and lung; and (5) patients discharged or transferred to hospital automatically. The patients were divided into the control group (58 patients) and the observation group (58 patients) according to the random number table. The control group received routine nursing care, while the observation group received cluster nursing based on risk management strategy. There were 33 males and 25 females in the control group. Their age ranged from 38 to 68 years, with an average of 49.51 ± 6.13 years. The Acute Physiology and Chronic Health Evaluation II (APACHE II) scores were 14–26 points, with an average of 18.13 ± 2.81. There were 34 males and 24 females in the observation group. Their age ranged from 32 to 67 years, with an average of 48.52 ± 5.91 years. The APACHE II scores ranged from 13 to 27, with an average of 18.05 ± 2.69. There was no statistically significant difference in general information between the two groups, which was comparable (*p* > 0.05).

### Research Methods

#### Control Group Using Traditional Nursing Methods

Nurses strengthened safety nursing work and carried out comprehensive health knowledge education to patients and their families. Strictly carry out aseptic operation, wash your hands, and wear sterile gloves when inserting a urinary catheter. Select an appropriate type of catheter and perform a gentle insertion process. After the urinary catheter was placed, the balloon was used for routine fixation and the perineum was rinsed with warm water twice a day. Keep the position of the urine collection bag always lower than the level of the bladder and ensure the patency of the closed drainage. Do a good job of patient restraint, set guardrail beside the bed, and regularly check whether the power supply of medical devices is in good condition to avoid accidents.

#### Observation Group Using Cluster Nursing Based on Risk Management Strategy

##### Risk Management Strategy, Cluster Nursing Plan Formulation, and Training

Before the cluster nursing based on risk management strategy was implemented, the infection management department trained the medical staff and an infection control group related to indwelling urinary catheter was established in the department. The group members included the department director, head nurse, and one physician and nurse. Through consulting relevant literature in databases such as Wanfang Database and HowNet Database, intervention measures provided by each guide, expert consensus, and research literature were analyzed. According to the condition, treatment characteristics and susceptible factors of patients with severe craniocerebral injury, risks, and potential risk factors in daily work were analyzed and identified, including gender, age, type of comorbidity (diabetes, etc.), types of antibiotics used, indwelling catheter time, hospitalization time, etc., and then CAUTI was summarized, organized, discussed, and defined one by one. Targeted prevention and control measures for CAUTI were formulated and training was conducted on time of indwelling urinary catheter and time of catheter removal. Department director and head nurse urged team members to strictly implement the CAUTI risk prevention and control measures, irregular supervision and inspection, and continuous improvement of prevention and control measures.

##### Specific Measures of Cluster Nursing Based on Risk Management Strategy

The responsible nurses performed risk assessment on the patients with indwelling urinary catheter and given antibiotic-coated urinary catheter. (1) Standardizing urethral catheterization method: The maximum sterile barrier was provided and a large sterile towel was laid when the catheter was placed. Hand hygiene was well done. The seven-step washing technique was used before and after nursing the catheter part or operating the urethral catheterization device. The sterile operation was strictly performed and the placing of the catheter was performed gently; (2) Maintaining the tightness of the drainage system and secondary fixation: A three-cavity silica gel urinary catheter with appropriate thickness and a high-capacity reflux-proof mother–child urine collection bag were selected. In addition to balloon fixation, the secondary fixation was performed with a homemade urinary catheter patch. The urinary catheter was uniformly fixed on the penis for men and on the medial thigh for women; (3) Shortening the indwelling time of urinary catheter: A prereporting system was established after the intubation. The CAUTI high-risk “red and blue” logo was placed at the head of the bed of patient and observed every 2 h including urine volume, color, nature, patency of the urinary catheter, and perineal skin condition. Patients with catheter obstruction should be promptly dredged and replaced. Sterile operation should be strictly performed and physiological bladder irrigation should be given priority. Risk assessment was conducted for each shift and it was included as one of the contents of shift change. Everyday, when nurses change shifts, they check urethral catheterization of patients and make CAUTI risk assessment, which was one of the contents of the shifts. The assessment includes whether it is necessary to continue indwelling catheter. If removal is required, the nurse should set a reminder to remove the catheter without indwelling indication as soon as possible; and (4) Improving self-cleaning consciousness of patient: Communication and health education with families of patients were strengthened, so that they could participate in nursing care together. The patients were encouraged to drink about 3 L of water per day, keep the urine volume above 2 L/day, and take urine samples for etiological examination when necessary. For patients with fecal incontinence, disinfection in time after cleaning is essential.

##### Quality Control of Cluster Nursing Based on Risk Management Strategy

The department director or the head nurse inspects the number of new patients, the total number of hospitalized patients, the number of indwelling catheter, catheter days, and patient-specific information, including gender, age, disease type, catheter time, is expected to pull tube time, etc., truthfully check records, patrol the measures to carry out the situation, and timely guidance to the operation, theory, etc. A special inspection occurs once a week. The existing problems in nursing were analyzed and group members were organized to discuss and propose solutions.

### Observation Indicators

(1) The total incidence of CAUTI in patients between the two groups and the incidence of CAUTI in different time periods were compared. The diagnosis of CAUTI was as follows: the patient developed fever, chills, frequent micturition, urgent urination, pain or tenderness above pubic bone, and purulent discharge at urethral meatus 48 h after the indwelling urinary catheter. The laboratory tests showed an increase in blood white cells as well as red and white cells in urine routine test. The colony number in bacteriological culture of middle urine was ≥103 colony-forming unit (cfu)/ml (15).
(2) The results of bacterial culture on the surface of urinary catheter were compared between the two groups before and after cleaning of urinary catheter as well as after 6 and 12 h of cleaning. Bacterial culture was performed on the outer end of the urinary catheter. Surface sampling method: The outer end of the urinary catheter was coated with a cotton swab with a length of 12.5 cm (the circumference and length of the urinary catheter were 2 and 12.5 cm, respectively) and the surface area of the outer end of the urinary catheter was close to 5 × 5 cm. The cotton swab was sent for testing in 10 ml of sampling solution and the number of colonies on the surface of the object was detected.
(3) The duration of indwelling urinary catheter, hospitalization, and hospitalization expenses were compared between the two groups.
(4) The incidence rates of nursing risk events such as pipeline falling off and nurse–patient disputes during the treatment were compared between the two groups.
(5) Results of midstream urine culture in patients with CAUTI are performed.
(6) The satisfaction scores of the two groups of patients were compared with the satisfaction questionnaire prepared by the department. At the time of discharge, the head nurse distributed to the patients a nursing satisfaction questionnaire about catheterization, which was divided into four options: very satisfied, satisfied, dissatisfied, and very dissatisfied. The scale adopts a percentage system, with >90 being very satisfied, 76–90 being satisfied, 60–75 being dissatisfied, and <60 being very dissatisfied.
(7) The knowledge, attitude, and practice (KAP) scale for prevention of catheter infection and the evaluation scale of nurses ability were used to evaluate the sense of control ability and core ability of nurses. The KAP scale for prevention of catheter infection included four dimensions including attitude, behavior, indication of catheter placement, and infection prevention strategy and it consisted of 71 items, with 1–5 points for each item. The higher the score was, the better the perception and control ability was. The evaluation scale of nurses ability included three dimensions of emergency ability, health education ability, and communication ability and there were 80 items in total, with 0–4 points for each item. The higher the score was, the better the prompting ability was.

### Statistical Methods

The SPSS software version 22.0 (SPSS Incorporation, Chicago, Illinois, USA) was used for processing. The measurement data of the experimental data were expressed as mean ± SD (X̄± s) and the *t*-test was used for pairwise comparison. The count data were expressed as (%) and the comparison was performed using the chi-squared test. The test level was α = 0.05 and *p* < 0.05 indicated that the difference was statistically significant.

## Results

### Comparison of Total CAUTI Incidence Between the Two Groups and CAUTI Incidence in Different Time Periods

**Figure 1:**
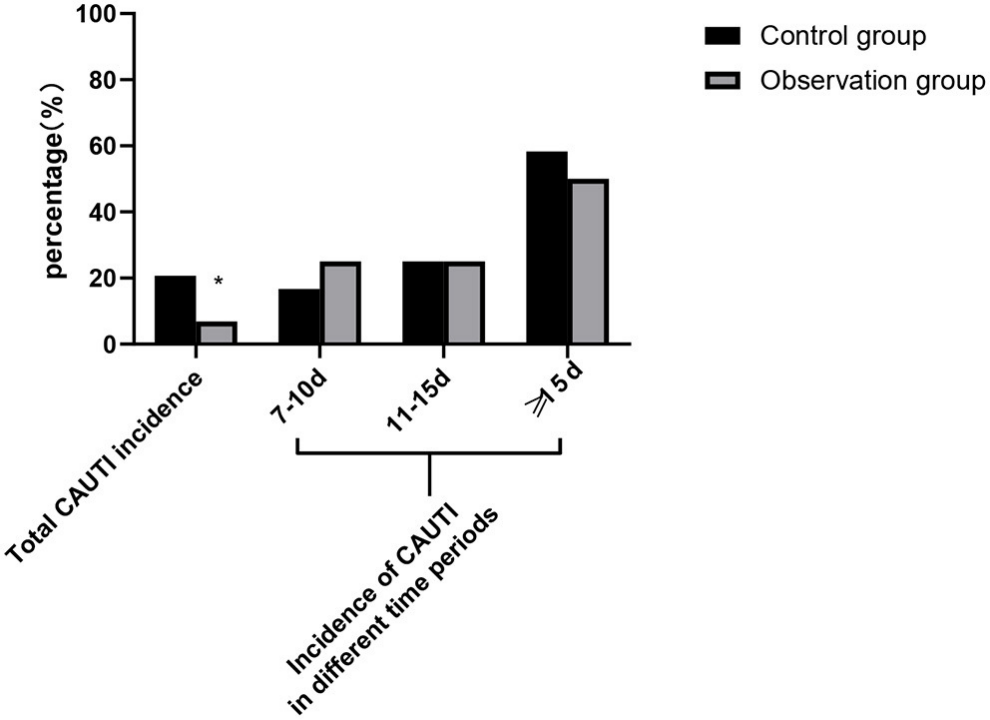
Comparison of total catheter-associated urinary tract infection (CAUTI) incidence between the two groups and CAUTI incidence in different time periods. Compared with the control group, *p < 0.05.

The total incidence rate of CAUTI in the control group was 20.69% including two patients with an indwelling urinary catheter for 7–10 days, three patients with an indwelling urinary catheter for 11–15 days, and seven patients with an indwelling urinary catheter above 15 days. The total incidence rate of CAUTI in the observation group was 6.90% including one patient with an indwelling urinary catheter for 7–10 days, one patient with an indwelling urinary catheter for 11–15 days, and two patients with an indwelling urinary catheter above 15 days. The total incidence of CAUTI in the observation group was lower than that in the control group and the difference was statistically significant (*p* < 0.05), as shown in Figure 1.

### Bacterial Culture Results on Catheter Surface of Patients in the Two Groups

**Figure 2:**
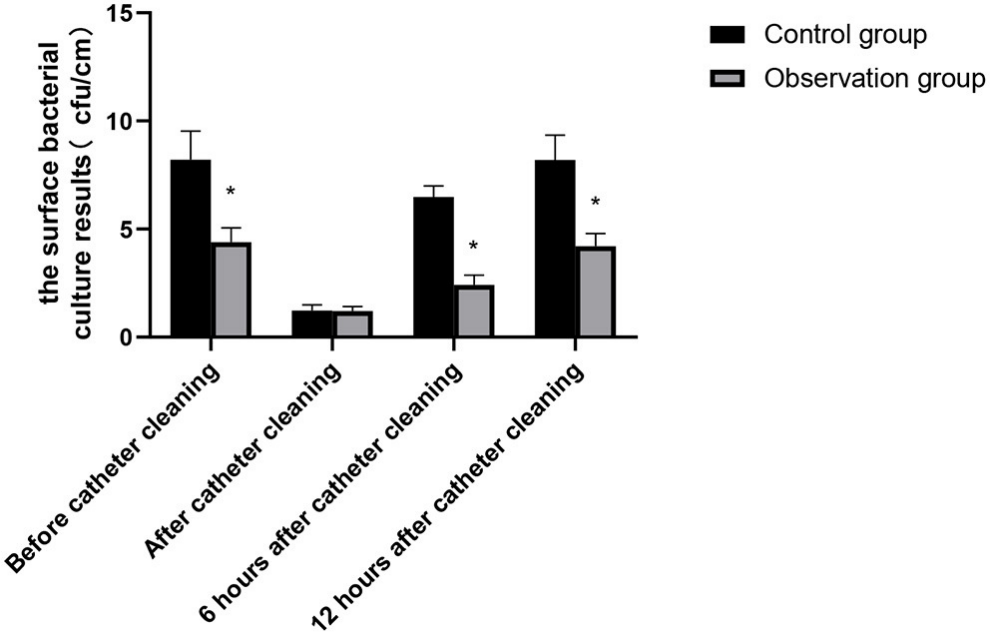
Bacterial culture results on catheter surface of patients in the two groups. Compared with the control group, *p < 0.05.

There was no significant difference in the surface bacterial culture results after catheter cleaning between the two groups (*p* > 0.05). The surface bacterial culture results on the catheter surface of patients in the observation group before and after 6 and 12 h of catheter cleaning was better than those of patients in the control group and the differences were statistically significant (*p* < 0.05), as shown in Figure 2.

### Comparison of Duration of Indwelling Urinary Catheter, Hospitalization Time, and Hospitalization Expenses Between the Two Groups

The duration of indwelling urinary catheter, hospitalization time, and hospitalization expenses of patients in the observation group was lower than those of patients in the control group and the differences were statistically significant (*p* < 0.05), as shown in Table 1.

**Table 1:** Comparison of duration of indwelling urinary catheter, hospitalization time, and hospitalization expenses between the two groups.

**Group**	***n***	**Duration of indwelling urinary catheter (d)**	**Hospitalization time (d)**	**Hospitalization expenses (yuan)**
Control group	58	19.76 ± 3.21	29.71 ± 4.62	33,892.76 ± 672.43
Observation group	58	15.92 ± 3.19	23.95 ± 4.15	31,527.52 ± 567.27
*t* value		6.462	7.064	20.475
*P* value		<0.001	<0.001	<0.001

### Comparison of the Incidence of Nursing Risk Events During Treatment Between the Two Groups

In the control group, there were one patient of urethral injury, three patients of urinary leakage, two patients of pipeline dropping out, and one patient of nurse–patient dispute during the treatment. The incidence rate of nursing risk events was 11.86%. In the observation group, there were one patient dropped out of the pipeline during the treatment and no nurse–patient dispute occurred. The incidence of nursing risk events was 1.72%. The incidence of nursing risk events in the observation group was lower than that in the control group. The difference was statistically significant (*p* < 0.05), as shown in Table 2.

**Table 2:** Comparison of the incidence of nursing risk events during treatment between the two groups.

**Group**	***n***	**Urethral injury**	**Urine leakage**	**Pipeline dropping out**	**Nurse-patient dispute**	**Incidence of nursing risk events**
Control group	58	1 (1.72%)	3 (5.18%)	2 (3.45%)	1 (1.72%)	12.07%
Observation group	58	0 (0.00%)	0 (0.00%)	1 (1.72%)	0 (0.00%)	1.72%
*χ^2^* value						4.721
*P* value						0.030

### Middle Urine Culture Results of Patients With CAUTI

A total of 16 patients with CAUTI were cultured in clean midstream urine. The cultured bacteria were: *Escherichia coli* (*E. coli*) (62.50%), *Enterococcus faecalis* (25.00%), and *Proteus* (12.50%) in descending order.

### Comparison of Patient Satisfaction Scores Between the Two Groups

**Figure 3:**
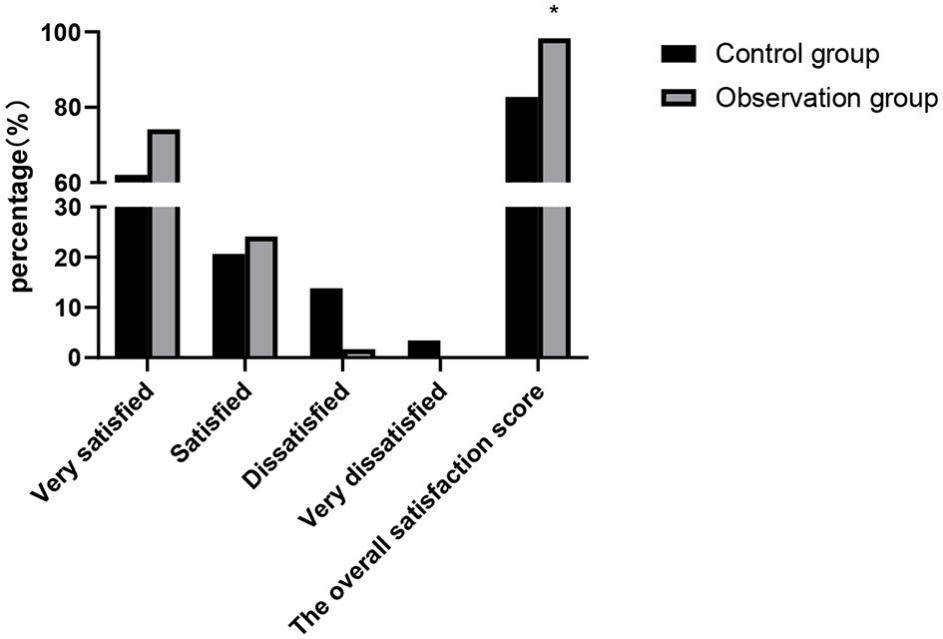
Comparison of patient satisfaction scores between the two groups. Compared with the control group, *p < 0.05.

The comparison with the self-made satisfaction questionnaire showed that the overall satisfaction score of the observation group was higher than that of the control group and the difference was statistically significant (*p* < 0.05), as shown in Figure 3.

### Nursing Staff Sense Control Ability and Core Ability Comparison Between the Two Groups

**Figure 4:**
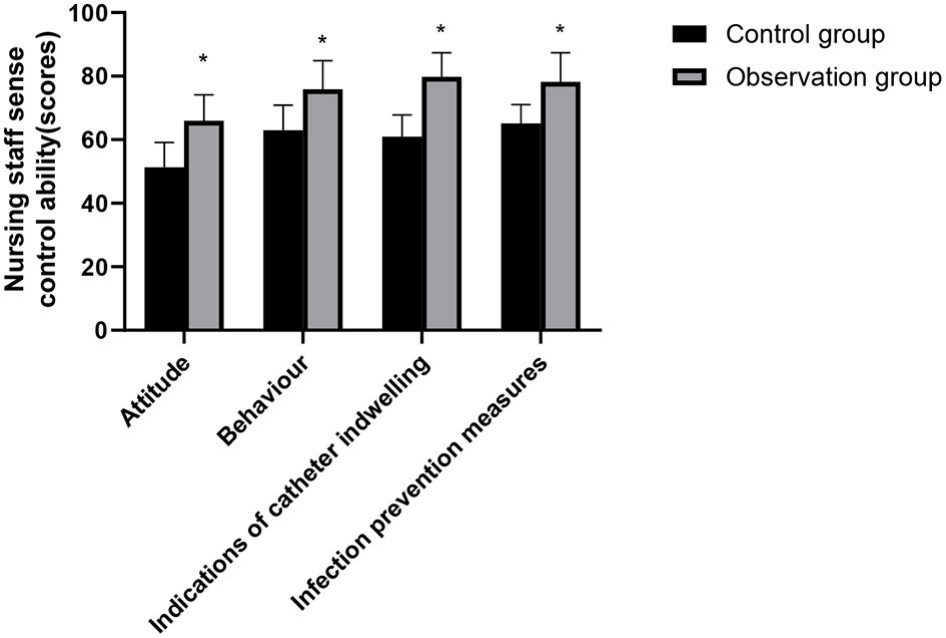
Nursing staff sense control ability comparison between the two groups. Compared with the control group, *p < 0.05.

**Figure 5:**
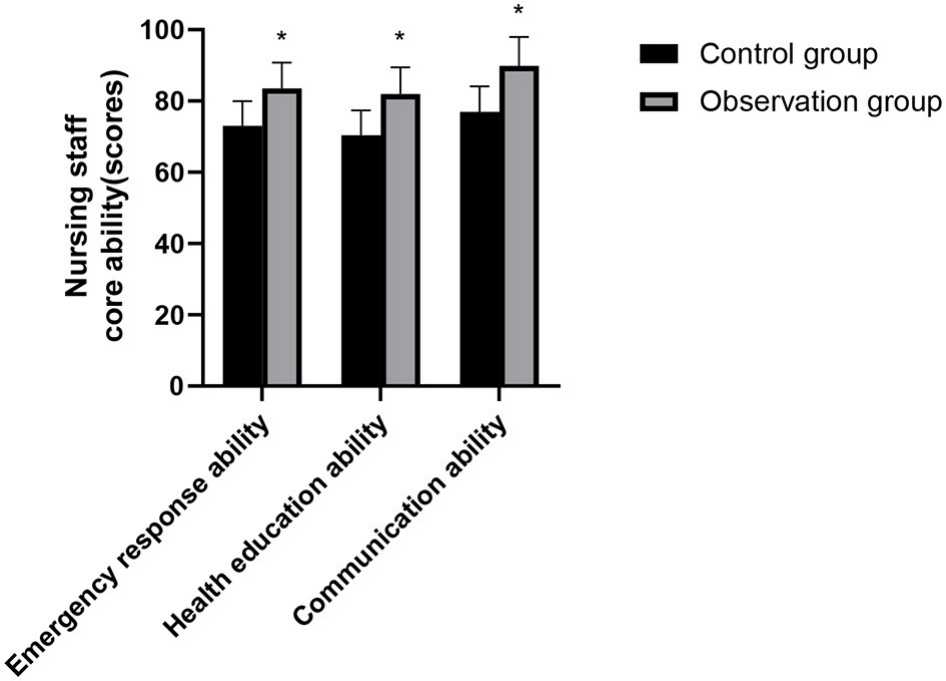
Nursing staff core ability comparison between the two groups. Compared with the control group, *p < 0.05.

The scores of core competencies such as attitude, behavior, indication of catheter indwelling and infection prevention measures, emergency response ability, health education ability, and communication ability of the nursing staff in the observation group were higher than those of the nursing staff in the control group and the differences were statistically significant (*p* < 0.05), as shown in Figures 4, 5.

## Discussion

Operation is the main treatment method for patients with severe craniocerebral injury. Although it can reduce the pain of patients, the catheter needs to be indwelling for a long time after surgery. Naturally, bacteria will grow after 1 week of indwelling urethral catheter in patients. With the extension of the indwelling time, the occurrence rate of bacterial biofilm is higher and higher and the formed biofilm gradually diffuses inside and outside the catheter surface through the lumen. This, in turn, leads to UTI, catheter blockage, and other conditions, seriously affecting the surgical effect and the recovery process of the postoperative patients (16–18).

In the recent years, cluster nursing has developed into a new concept of clinical nursing. Compared with the traditional nursing mode, it has the systematic characteristics and can carryout comprehensive intervention on the disease, which is more effective than performing a single nursing measure alone (19). Cluster nursing based on risk management strategy includes risk identification and evaluation, implementation of specific nursing measures, and nursing quality control. Risk identification and risk assessment are the first step, which can identify the objective existence and potential risk factors of CAUTI, comprehensively and quantitatively assess the risk of patients with CAUTI, and provide reference for the subsequent formulation and implementation of specific nursing measures. Nurse quality control is that last link of nursing intervention and also the key step to avoid adverse event and improve nursing quality.

In this study, the total incidence of CAUTI in the observation group was significantly lower than that in the control group and the incidence of CAUTI in both the groups increased gradually with the prolongation of indwelling urinary catheter. It is suggested that cluster nursing based on risk management strategy could reduce the incidence of CAUTI in patients with severe craniocerebral injury. Through consulting relevant literature and analyzing the interventions provided by various guidelines, expert consensus, and research articles, we found that the main risk factors of CAUTI were as follows: (1) Patient factors: Severe craniocerebral patients were in a coma, incontinent, the normal physiological environment of the urethral meatus and mucosa was imbalanced, the antibacterial function of neutrophils was weakened and destroyed, and the body resistance was decreased. In addition, the catheter indwelling time was long, which was more likely to lead to infection. However, own factors of patient, such as old age, basic diseases, and the severity of the disease, could not be interfered; (2) Personnel factors: The concept of sterility of medical staff is not strong, disinfection and isolation measures are not in place, and the compliance of hand hygiene is poor. Catheter placement technology is not skilled, the operation is not standardized, catheter care is not in place, etc.; and (3) Environmental factors: Family members of patients paid more visits, which easily brought pathogenic bacteria into the ward; pathogens can also be transmitted, if the daily environment is not cleaned properly (20–22). In view of the above risk factors, targeted prevention and control measures of CAUTI were formulated and strictly controlled for implementation to ensure the quality of care.

Based on the risk management strategy of cluster nursing, the specific implementation measures include: (1) Specification catheterization method, do a good job in hand hygiene, and strict aseptic operation; (2) Suitable urinary catheter was selected and self-made urinary catheter paste was used for secondary fixation; (3) To solve the problem of the long indwelling catheter in patients, setup risk assessment and reminder for indwelling catheter, so as to remove it as soon as possible; (4) Strengthen communication with families of patients and health education of cleaning and disinfection. Clustering consisting of four elements is feasible for every nursing measure in clinic. The results of this study showed that the bacterial culture results on the surface of urinary catheter of patients in the observation group in different time periods were better than those of patients in the control group. At the same time, the time of indwelling urinary catheter, hospital stay, and hospital expenses of patients in the observation group were lower than those of patients in the control group. Moreover, the incidence of nursing risk events was also significantly reduced. A study by Mitchell showed that the incidence of CAUTI was positively correlated with the catheter indwelling time and the longer the catheter indwelling time, the more bacteria colonization outside the catheter (23). The time of indwelling urinary catheter for patients in the observation group was shortened. By setting the reminder mechanism, the doctors were prompted to stop pulling out the urinary catheter according to the advice of doctor in time, so as to improve the effectiveness of nursing implementation. In this study, we performed secondary fixation of the urinary catheter to avoid the change in the position of the urinary catheter and ensure that no mechanical damage was caused to the urethra and bladder mucosa during the period of indwelling urinary catheter, thereby reducing the occurrence of CAUTI.

A total of 16 patients with CAUTI were cultured in clean midstream urine and the cultured strains were in the order of *E. coli* > *Enterococcus faecalis* > *Proteus* from high to low. *E. coli* in the Gram-negative Enterobacteriaceae family is a common pathogen isolated from the urinary tract. A study covering 38 centers in 11 countries in the Asia-Pacific region showed that the constituent ratio of *E. coli* in Gram-negative bacterial isolates causing UTI was 56.8% (24).

With the increasing awareness of hospital infection monitoring and demand of people for medical services, the incidence of medical disputes has been increasing year by year. It is an inevitable trend of the management development of medical institutions in the future to reduce the infection factors from the root, reduce the incidence of infection, ensure the safety of patients, and improve the effect of care details and medical quality (25). In this study, the overall satisfaction score of patients and the control and core ability scores of nursing staff in the observation group were higher than those in the control group. It proved the high-quality application effect of cluster nursing based on risk management strategy in the process of nursing risk management for patients with severe craniocerebral injury. Nursing can assess the risk of nursing by mastering the characteristics of condition of patients and summarize the high-risk factors and the advantages and disadvantages of intervention measures, which will help to improve the quality of clinical nursing and reduce the incidence of nursing risk events. At the same time, it can improve the subjective ability of nursing staff to resist risks, the quality of nursing care, and the degree of patient care satisfaction (26).

In summary, cluster nursing based on risk management strategy can effectively reduce the incidence of nursing risk events and the probability of UTI in the management of UTI in patients with severe craniocerebral injury, shorten the duration of indwelling urinary catheter and hospitalization, and improve the nursing satisfaction and the sense of control and nursing ability of nursing staff.

## Data Availability Statement

The original contributions presented in the study are included in the article/supplementary material, further inquiries can be directed to the corresponding author.

## Ethics Statement

The studies involving human participants were reviewed and approved by the Ethics Committee of Chongqing Southeast Hospital. Written informed consent to participate in this study was provided by the participants' legal guardian/next of kin.

## Author Contributions

HQ, JY, and CW: collect datas. HQ: write the paper. CW: revise the paper. All authors contributed to the article and approved the submitted version.

## Conflict of Interest

The authors declare that the research was conducted in the absence of any commercial or financial relationships that could be construed as a potential conflict of interest.

## Publisher's Note

All claims expressed in this article are solely those of the authors and do not necessarily represent those of their affiliated organizations, or those of the publisher, the editors and the reviewers. Any product that may be evaluated in this article, or claim that may be made by its manufacturer, is not guaranteed or endorsed by the publisher.
